# Incidence and Risk Factors for Arterial Thrombosis in Patients with Acute Leukemia and Lymphoid Malignancies: A Retrospective Single-Center Study

**DOI:** 10.3390/cancers16142511

**Published:** 2024-07-10

**Authors:** Jenna Hellman, Roza Chaireti

**Affiliations:** 1 Department of Molecular Medicine and Surgery, Karolinska Institute, 17177 Solna, Sweden; jenna.k.hellman@gmail.com; 2 Department of Hematology, Karolinska University Hospital, 17176 Stockholm, Sweden; 3 Department of Medicine, Karolinska Institute, 17177 Solna, Sweden

**Keywords:** acute leukemia, lymphoma, chronic lymphatic leukemia, arterial thromboembolism

## Abstract

**Simple Summary:**

While a lot is known about the role of cancer in the development of blood clots in the veins, little is known about the way cancer affects the risk of developing blood clots in the arteries, such as in the brain (stroke) and the heart (infarction). Our study aims to evaluate the risk of developing arterial blood clots in patients with blood cancer (acute leukemia and lymphoid malignancies). Although we found that those patients do not have a particularly high risk of developing thrombosis, we did discover that they had risk factors for developing clots and that the arterial clots were frequently diagnosed at the same time as the cancer, which can complicate the initiation of chemo-therapy. Our research contributes data to an area where data are lacking, and can guide physicians when they make decisions on the potential discontinuation of blood thinners, which are instrumental in preventing arterial clots.

**Abstract:**

Introduction: The treatment of patients with hematological malignancies and acute arterial thrombosis (ATE) is challenging due to the risk of bleeding complications during treatment. Data on the incidence and risk factors for ATE in this group are very limited. Aims: We aimed to evaluate the incidence and risk factors for ATE in patients with acute leukemia (AL) and lymphoid malignancies. Material and Methods: Patients with acute leukemia (AL), lymphoid malignancies, and ATE diagnosed following cancer diagnosis, who were treated and followed at the Department of Hematology, Karolinska University Hospital, 2005–2020, were candidates for inclusion in this study. Retrospective data on malignancy, ATE, and risk factors were collected. Results: No differences in either the 15-year incidence of ATE (1.4%) nor in the risk factors for cardiovascular disease (CVD) between patients with AL and lymphoid malignancies and ATE were found. ATE at diagnosis was more frequent in patients with AL and lymphoid malignancies (excluding chronic lymphatic leukemia, CLL). Conclusions: Patients with AL and lymphoid malignancies have a similar risk of ATE when compared to each other and the general population, regardless of platelet levels. No difference could be found in the presence of CVD risk factors between patients with AL and lymphoid malignancies presenting with ATE.

## 1. Introduction

Even though venous thromboembolism in patients with cancer is well studied, data on arterial thromboembolism (ATE) are limited. ATE in this patient group has been associated with a 3.2–5-times higher risk of mortality compared to cancer patients without ATE [1]. The risk of ATE is highest during the first 12 months after cancer diagnosis and during active cancer treatment [1,2]. Patients with lung, prostate, bladder, or kidney cancer, or with advanced stage cancer (stages III and IV), have the highest risk of developing ATE [3,4,5]. Other variables associated with an increased risk of ATE are age, male gender, smoking, having comorbidities (i.e., infection, pulmonary disease, hypertension (HT), hyperlipidemia (HL), diabetes mellitus type 2 (DM2), congestive heart failure, etc.) [4,5], as well as certain chemotherapeutic agents (angiogenesis inhibitors, alkylating agents, antimetabolites, antimicrotubules, proteasome, and aromatase inhibitors [6]) and tamoxifen, L-asparaginase, all-trans-retinoic acid, erythropoietin, and radiotherapy [1,7,8,9]. The underlying mechanisms include vascular toxicity, endothelial dysfunction, platelet aggregation, reduced levels of nitrous oxide, and vasospasm [6].

Hematological malignancies are a heterogenous group of cancers with varied etiology and are associated with increased risk for venous and, to a lesser extend, arterial thromboembolism [7,10]. Adult patients with hematological malignancies have been shown to have an increased risk primarily of acute myocardial infarction, but also of ischemic stroke, compared with the general population [10], but the data is scarce. Thrombocytopenia has not been associated with a reduced risk of recurrent or first-time ATE in patients with hematological malignancies [11], and the prevention and treatment of ATE is challenging in patients with hematological malignancies due to an elevated risk of bleeding complications, mainly because of the presence of disease- and treatment-associated thrombocytopenia, leading to major or even fatal bleedings [12].

Our study aims to contribute with evidence in this area with inadequate data by reviewing the medical records of patients with acute leukemia (AL: acute myeloid leukemia, AML, and acute lymphoblastic leukemia, ALL) and lymphoid malignancies who were treated at the Department of Hematology, Karolinska University Hospital, Stockholm, Sweden, between 2005 and 2020, and who suffered from ATE following cancer diagnosis. We studied differences in the incidence and risk factors for ATE in these two patient groups.

## 2. Materials and Methods

### 2.1. Study Design and Patients

In this retrospective, non-interventional cohort study, patients with AML, ALL, and lymphoid malignancies (including chronic lymphatic leukemia, CLL) who were treated at the department of Hematology at Karolinska Hospital, Stockholm, between January 2005 and August 2020, and who were diagnosed with ATE following a diagnosis of hematological cancer, and while having an active disease, were eligible for inclusion. These patients were identified by using the International Classification of Diseases tenth revision (ICD-10) codes for AML, ALL, lymphoid malignancies, and ATE, and the end points for the data collection were death, complete remission, or 31 August 2020.

This study was approved by the Ethical Review Board in Stockholm, Sweden.

### 2.2. Collected Data

Patients were identified by using ICD-10 codes and data was collected by reviewing the patients’ digital medical records, including free-text searches.

All ATE events were reviewed, and patients with type 2 myocardial infarctions or hemorrhagic strokes were excluded. In unclear cases, the events were classified as myocardial infarction (*n* = 5) and ischemic stroke (*n* = 3), and were included as ATE, if the patients had been treated according to the protocols of the respective ATE.

Demographic data (age and sex), data on hematological disease (date of diagnosis, type, treatment, and response to treatment), and data on ATE (date of diagnosis and type), as well as associated risk factors (age, DM2, HT, HL, body mass index (BMI), atrial fibrillation (AF), smoking, prior ATE, ongoing systemic or pulmonary infection, and ongoing cancer or antiplatelet treatment) and previous treatment of risk factors (i.e., serum lipid, blood sugar or blood pressure lowering agents), were recorded.

Since a hematological malignancy is most likely present at least a month before the diagnosis is confirmed, all ATE and bleeding events taking place 30 (±5) days prior to cancer diagnosis were included in this study. ATE was judged to have occurred at the time of initial diagnosis of AL or upon the diagnosis of lymphoid malignancies (presenting manifestation, not associated with treatment) if it occurred within one month prior to or four days after the cancer diagnosis, and thus before the initiation of any chemotherapeutic agent.

Ongoing cancer treatment was defined as any treatment of the hematological malignancy at the date of ATE diagnosis. Antiplatelet or anticoagulant treatment was defined as ongoing if it was discontinued, at the earliest, one day before ATE or, in the case of warfarin, three days prior to an event.

### 2.3. Statistical Analysis

Descriptive statistics were used to present the data on ATE and risk factors within the patient groups. Chi-square tests and Fisher’s exact tests for *p* values were used to evaluate the effect of different parameters on the outcomes between the patient groups. All reported *p*-values were two-sided. IBM SPSS Statistics 27 software was used for statistical analysis. A value of *p* < 0.05 was considered statistically significant. All percentages were calculated based on the intention-to-treat i.e., the total number of included patients, including patients with missing values.

## 3. Results

### 3.1. Patient Population

In total, 67 patients, of which 16 (23.9%) were diagnosed with AL and 51 (76.1%) with lymphoid malignancies, with 18/51 (32.30%) patients with CLL, were included in this study. Of the 33 patients with a lymphoid malignancy other than CLL, 22 had B-cell lymphoma, 7 had T/NK-cell lymphoma, 2 had follicular lymphoma, and 2 had Hodgkin lymphoma. Among patients with AL, 3/16 (18.8%) were diagnosed with ALL, and the rest with AML. Due to the small number of patients with AL, no subgroup analyses were performed for this group. The mean age for the patients at the diagnosis of hematological cancer was 69.3 ± 9.5 years (SD), and 46/67 (68.7%) were male. The characteristics of patients presenting with ATE are presented in Table 1.

The demographic and other characteristics of the cohort are shown in Table 1.

### 3.2. Incidence of ATE

Altogether, 67 patients with AL and lymphoid malignancies were diagnosed with ATE following cancer diagnosis. During the study period of 2005–2020, 1100 patients with AL (158 with ALL and 942 with AML) and 3586 patients with lymphoid malignancies were treated at the Karolinska University hospital in Stockholm. The 15-year incidence of ATE was 1.4%, which was similar for both groups.

A total of 11/67 (16.4%) ATEs occurred at the time of cancer diagnosis but prior to the initiation of cancer treatment. About one-third of all ATEs, 22/67 (32.8%), were diagnosed within 3 months, 30/67 (44.8%) within 6 months, and the majority (36/67, 53.7%) within a year of cancer diagnosis, but prior to remission.

Of all the patients with ATE after cancer diagnosis, roughly one-third (23/67, 34.3%) had a history of previous ATE prior to cancer diagnosis. ATE was the cause of death in 14/61 (23.0%) of the recorded deaths in patients with ATE after cancer diagnosis.

### 3.3. Risk Factors for ATE

In patients with ATE, the most frequent risk factors present were age ≥ 65 years (57/67, 85.1%), hypertension (45/67, 67.2%), overweight (39/67, 58.2%), and smoking (37/67, 55.2%), followed by hyperlipidemia (29/67, 43.3%) and DM2 (10/67, 14.9%). In average, each patient with ATE after cancer diagnosis had 3.2 risk factors for CVD. There was no significant difference between patients with AL, CLL, and other lymphoid malignancies in relation to cardiovascular risk factors or the type of ATE, age, or history of ATE, or the presence of infection at the time of the ATE. The risk factors and other characteristics of ATE episodes are presented in Table 2.

### 3.4. ATE in Patients with Acute Leukemia

The 15-year-incidence of acute myocardial infarction and stroke among patients with lymphoid malignancies was 1.2% and 0.3%, respectively. No other type of ATE event was recorded.

Of the sixteen patients with acute leukemia and ATE, eight (50%) had a history of ATE before cancer diagnosis. Significantly more patients with AL had ATE while having thrombocytopenia, compared to patients with lymphoma, where 87.5% of ATEs occurred when platelets were <150 × 10^9^/L (*p* < 0.001) and 50.0% during severe thrombocytopenia (<50 × 10^9^/L, *p* = 0.001) in patients with AL.

### 3.5. ATE in Patients with Lymphoma and CLL

The 15-year-incidence of acute myocardial infarction and stroke among patients with lymphoid malignancies was 0.8% and 0.5%, respectively, whereas the incidence of other ATE events (such as peripheral embolism, etc.) was 0.08%.

Altogether, 51 patients with lymphoid malignancies, of which 18 had CLL, had an ATE after cancer diagnosis. In patients with lymphoma, the mean time from cancer diagnosis to ATE was 1.8 years, whereas for patients with CLL, it was 9.6 years. In five patients with lymphoma, the time from diagnosis to ATE was exceptionally long, at 3.5–4 years. These included two patients with mycosis fungoides and three with indolent lymphomas (follicular, marginal zone, and B-cell lymphoma).

Among the patients with ATE after cancer diagnosis, one-third (10/33, 30.3%) of the patients with lymphoma and 5/18 (27.8%) patients with CLL had been diagnosed with an ATE prior to cancer diagnosis. The majority (31/51, 60.7%) of ATE cases in patients with lymphoma and CLL occurred when the platelet count was normal, and in two cases (3.7%) during severe thrombocytopenia (platelets <50 × 10^9^/L).

## 4. Discussion

This study aimed to evaluate the incidence and risk factors of ATE in a cohort of patients with AL and lymphoid malignancies during a 15-year period. The 15-year-incidence was 1.4% and was similar in both groups. No differences were observed between those groups. Compared to lymphoid malignancies, patients with AL more frequently had ATE as a presenting manifestation at the time of cancer diagnosis or during treatment, as well as a lower platelet count at the time of an ATE event.

The majority of patients with ATE in this study were male (68.8%). Globally, men have a 1.5-times higher rate of ischemic heart disease compared to women [13], as well as a higher incidence for lymphoma and CLL [14,15]. In addition, male gender has been found to be an independent risk factor for ATE in patients with cancer [14]. This may explain the over-representation of men in our cohort.

Adelborg et al. reported a higher incidence rate of AMI in patients with AML and CLL, and of ischemic stroke in patients with Hodgkin lymphoma, in contrast to a reference cohort without cancer [10]. In our cohort, the 15-year incidence for both thromboembolic stroke and AMI was lower compared to the respective incidence in the general population (5% and 4.9%, respectively; data from the National Board of Health and Welfare) [16]. However, the data from the Swedish database [16] include even hemorrhagic stroke and do not distinguish between type 1 and type 2 AMI. It is therefore adequate to assert that the incidence for thrombotic stroke and AMI is lower in the general population; those data were not available to us at the time of the study, though. In addition, some patients with stroke and AMI in our cohort were treated for ATE in hospitals other than the Karolinska University Hospital. Due to the lack of a common medical records system in Stockholm County, especially during the early years of our study, it is possible that some events were missed. In a retrospective, one-center study, the 9-year-incidence of arterial thrombosis in patients with acute leukemia was 2/157 (1.3%), and in lymphoma patients it was 6/238 (2.5%) [2]. It is unclear why the incidence for ATE among patients with lymphoid malignancies was lower in our study. A contributing factor could be the centralization of the care of patients with lymphoid malignancies in Stockholm County during the study period, which could have led to missing such patients and ATE events, a more frequent usage of anticoagulants as primary stroke prophylaxis in patients with AF, or a higher usage of antiplatelet agents in patients with risk factors, even in the absence of prior thromboembolism. Those aspects were, however, not studied in this report.

In our cohort, the majority (63%) of ATE occurred within 12 months after cancer diagnosis, which is supported by other studies with similar designs and aims [1]. In patients with AL, ATE occurred at the time of diagnosis and before any cancer treatment in nearly half of the cases of all ATE, and this is in alignment with a previous study evaluating both VTE and ATE [17]. A low platelet count was not found to be a protective factor for ATE in thrombocytopenic cancer patients [11], and, although we did not perform any specific analyses, it was shown that ten patients suffered from ATE despite concurrent severe thrombocytopenia. Most patients with AL were diagnosed with ATE at the time when their platelet values were subnormal. This is not a surprising finding, considering the possible consumption thrombocytopenia in those patients.

According to local recommendations, and in accordance with international guidelines [18,19], antiplatelet agents should be discontinued when platelets are <50 × 10^9^/L and recommenced after platelets are >50 × 10^9^/L. A quality study (unpublished data) on all patients with AL and lymphoid malignancies with antiplatelet treatment during 2005 and 2020 in our center showed that, in almost 50% of cases, the recommendations were not followed. In most cases, treatment with antiplatelet agents was not recommenced following an increase in platelet count >50 × 10^9^/L, probably due to the expected recurrence of thrombocytopenia. However, in almost half of the patients with AL, ATE occurred at the time of the diagnosis, and significantly more patients with lymphoid malignancies other than often-indolent CLL similarly had ATE at diagnosis. This could be indicative of the association between the aggressive disease and ATE, highlighting the need to take risk factors into consideration when deciding on whether to withhold prophylaxis despite adequate platelet levels.

In this cohort, the patients had an average of 2.4 risk factors for CVD, but no statistically significant difference could be seen between patients with AL, CLL, and lymphoma. Considering the scarce previous data, it is hard to evaluate the accuracy of our findings. However, in the general population, 80–90% of patients presenting with initial myocardial infarction had at least one risk factor for coronary heart disease [20]. In addition, a history of smoking, DM2, and the use of lipid-lowering or anti-hypertensive medication have been associated with an increased risk of ATE in patients with cancer in an univariable analysis [4]. The consensus is that the prevention of ATE complications should focus on the optimal evidence-based individual management of known risk factors for CVD, including hypertension, DM2, hyperlipidemia, and prior ATE, as well as primary prevention.

The main strength of this study was that it was conducted at a single center with the same treatment regime both for hematological malignancies and ATE, and therefore any changes made to the treatment protocols applied to all patients. Another strength was that we used free-text search in patient records to find ATE events. This has resulted in a more complete list of outcomes. An additional strength was that we reviewed each ATE event, and only those fulfilling the inclusion criteria were evaluated in this study.

The main limitation is that this study was retrospective, and relevant data might therefore be missing. In addition, access to patient data was only available for the Karolinska University Hospital. Some patients have also been hospitalized or have died in other hospitals in Stockholm County, and, hence, the possibility of ATE as the cause of death for more patients is unknown. This could mean that the incidence of ATE after the cancer diagnosis, as well as the frequency of ATE as the cause of death, could be higher. However, we did include ATE events which were diagnosed at other hospitals, if those occurred prior to remission, since they were documented in the patients’ medical records upon follow-up. Another limitation of the current study was the lack of a matched reference cohort of onco-hematological cancer patients without ATE after diagnosis, which would allow for a more precise evaluation of the impact of the risk factors and comparisons regarding incidence.

## 5. Conclusions

With an increasing aging population, the incidence of cancer is rising among patients with cardiovascular risk factors because more patients are surviving for a longer time, even with incurable cancer, due to new treatment regimens and more advanced palliative options. There is need for more detailed knowledge on the risk and benefit profile of antiplatelet agent treatments, especially in patients with hematological malignancies with thrombocytopenia resulting from cancer and aggravated by chemotherapy. Even though our study did not show that patients with AL and lymphoid malignancies have an exceptionally high risk of ATE, in almost 50% of the cases, arterial events were diagnosed during the first six months following diagnosis. The concurrent treatment of ATE with chemotherapy treatment is challenging, and it is paramount to prevent such complications through rigorous primary prophylaxis and through avoiding withholding antiplatelet agents when unnecessary.

## Figures and Tables

**Table 1:** Characteristics of patients with acute leukemia and lymphoid malignancies treated at Karolinska University Hospital, January 2005–August 2020, and diagnosed with ATE. Significant *p*-values in bold (*p* < 0.005). Values presented as *n* (%) if not otherwise stated.

	AL *n* = 16	Lymphoid Malignancy *n* = 51			*p*-Value		
	**(1)**	**(2)**	**CLL *n* = 18 (2a)**	**Lymphoma *n* = 33 (2b)**	**1 and 2**	**1 and 2a and 2b**	**1 and 2b**
Age (mean ± SD) at diagnosis	72.6 ± 11.7	68.1 ± 9.7	64.3 ± 10.4	70.1 ± 8.8			
ATE at diagnosis	7 (43.8%)	4 (7.8%)	0	4 (12.1%)	0.001	0.002	0.013
Gender					0.993	0.582	0.724
Male	11 (68.8%)	35 (68.6%)	14 (77.8%)	21 (63.6%)			
Female	5 (31.2%)	16 (31.3%)	4 (22.2%)	12 (36.4%)			
Age (mean ± SD) at ATE after diagnosis	73.5 ± 11.5	72.6 ± 8.8	73.9 ± 8.9	71.9 ± 8.8			
No. of deceased	14 (87.5%)	44 (86.3%)	16 (88.9%)	28 (84.8%)	0.900	1.000	1.000
Cause of death					0.300	0.468	0.282
ATE	5 (35.7%)	9 (20.5%)	4 (25%)	5 (17.9%)			
Hemorrhage	1 (7.1%)	1 (2.3%)	0	1 (3.6%)			
Other	3 (21.4%)	19 (43.2%)	5 (31.3%)	14 (50%)			
Unknown	5 (35.7%)	15 (34.1%)	7 (43.8%)	8 (28.6%)			

CLL: chronic lymphatic leukemia; ATE: arterial thromboembolism; MI: myocardial infarction; SD: standard deviation.

**Table 2:** Characteristics of ATE in patients with acute leukemia and lymphoma.

	Acute Leukemia *n* = 16	Lymphoid Malignancies *n* = 51 (2)			*p*-Value		
	**(1)**		**CLL, *n* = 18 (2a)**	**Lymphoma, *n* = 33 (2b)**	**1 and 2**	**1 and 2** **a and 2b**	**1 and 2b**
Type of ATE					0.287	0.249	0.107
MI	13 (81.3%)	29 (56.9%)	12 (66.7%)	17 (51.5%)			
Stroke	3 (18.7%)	19 (37.3%)	6 (33.3%)	13 (39.4%)			
Other	0	3 (5.9%)	0	3 (9.1%)			
Mean platelet value at ATE (×10^9^/L)	121.3 (11–567)	208.4 (5–512)	143.6 (5–253)	239.8 (50–512)	0.001 *	0.001 *	<0.001 *
History of ATE prior to cancer diagnosis	8 (50%)	15 (29.4%)	5 (27.8%)	10 (30.3%)	0.144	0.333	0.206
Cancer treatment at ATE	11 (68.6%)	22 (43.1%)	7 (38.1%)	15 (45.5%)	0.074	0.183	0.125
Hypertension	12 (75.0%)	33 (64.7%)	9 (50.0%)	24 (72.7%)	0.444	0.191	0.866
Hyperlipidemia	10 (62.5%)	19 (37.3%)	7 (38.9%)	12 (36.4%)	0.075	0.203	0.085
DM2	3 (18.8%)	7 (13.7%)	1 (5.6%)	6 (18.2%)	0.623	0.455	0.962
Smoking history	8 (50.0%)	29 (56.9%)	12 (66.7%)	17 (51.5%)	0.565	0.730	0.737
Age ≥ 65 years	14 (87.5%)	43 (84.3%)	16 (88.9%)	27 (81.8%)	0.755	0.906	0.614
BMI (kg/m^2^)					0.634	0.811	0.647
<25	5 (31.3%)	22 (43.1%)	8 (44.4%)	14 (42.4%)			
25–30	8 (50.0%)	19 (37.3%)	7 (38.9%)	12 (36.4%)			
>30	3 (18.7%)	9 (17.6%)	2 (11.1%)	7 (21.2%)			
AF (for patients with stroke)	2 (66.7%)	6 (31.6%)	1 (16.7%)	5 (38.5%)	0.527	0.327	0.550
Infection	7 (43.8%)	12 (23.5%)	2 (11.1%)	10 (30.3%)	0.117	0.102	0.354
Antithrombotics	3 (18.8%)	13 (25.5%)	5 (27.8%)	8 (24.2%)	0.556	0.870	0.627

CLL: chronic lymphatic leukemia; ATE: arterial thromboembolism; MI: myocardial infarction; DM2: diabetes mellitus type 2; BMI: body mass index; AF: atrial fibrillation. * Analyzed with platelet count divided into <150, 150–350, and >350. Statistically significant *p*-values in bold.

## Data Availability

Data are available upon request (and are saved at the Karolinska Institute ELN).

## References

[1] De Stefano V. (2018). Arterial thrombosis and cancer: The neglected side of the coin of Trousseau syndrome. Haematologica.

[2] Yıldız A., Albayrak M., Pala Ç., Afacan Öztürk H.B., Maral S., Şahin O., Cömert P. (2020). The incidence and risk factors of thrombosis and the need for thromboprophylaxis in lymphoma and leukemia patients: A 9-year single-center experience. J. Oncol. Pharm. Pract..

[3] Navi B.B., Reiner A.S., Kamel H., Iadecola C., Okin P.M., Elkind M.S., Panageas K.S., DeAngelis L.M. (2017). Risk of Arterial Thromboembolism in Patients With Cancer. J. Am. Coll. Cardiol..

[4] Grilz E., Königsbrügge O., Posch F., Schmidinger M., Pirker R., Lang I.M., Pabinger I., Ay C. (2018). Frequency, risk factors, and impact on mortality of arterial thromboembolism in patients with cancer. Haematologica.

[5] Khorana A.A., Francis C.W., Culakova E., Fisher R.I., Kuderer N.M., Lyman G.H. (2006). Thromboembolism in hospitalized neutropenic cancer patients. J. Clin. Oncol..

[6] Hassan S.A., Palaskas N., Kim P., Iliescu C., Lopez-Mattei J., Mouhayar E., Mougdil R., Thompson K., Banchs J., Yusuf S.W. (2018). Chemotherapeutic Agents and the Risk of Ischemia and Arterial Thrombosis. Curr. Atheroscler. Rep..

[7] Colombo R., Gallipoli P., Castelli R. (2014). Thrombosis and hemostatic abnormalities in hematological malignancies. Clin. Lymphoma Myeloma Leuk..

[8] Chotai P.N., Kasangana K., Chandra A.B., Rao A.S. (2016). Recurrent Arterial Thrombosis as a Presenting Feature of a Variant M3-Acute Promyelocytic Leukemia. Vasc. Specialist Int..

[9] Choudhry A., DeLoughery T.G. (2012). Bleeding and thrombosis in acute promyelocytic leukemia. Am. J. Hematol..

[10] Adelborg K., Corraini P., Darvalics B., Frederiksen H., Ording A., Horváth-Puhó E., Rørth M., Sørensen H.T. (2019). Risk of thromboembolic and bleeding outcomes following hematological cancers: A Danish population-based cohort study. J. Thromb. Haemost..

[11] Leader A., Gurevich-Shapiro A., Spectre G. (2020). Anticoagulant and antiplatelet treatment in cancer patients with thrombocytopenia. Thromb. Res..

[12] Kaatz S., Ahmad D., Spyropoulos A.C., Schulman S. (2015). Definition of clinically relevant non-major bleeding in studies of anticoagulants in atrial fibrillation and venous thromboembolic disease in non-surgical patients: Communication from the SSC of the ISTH. J. Thromb. Haemost..

[13] Wendelboe A.M., Raskob G.E. (2016). Global Burden of Thrombosis: Epidemiologic Aspects. Circ. Res..

[14] Siegel R.L., Miller K.D., Fuchs H.E., Jemal A. (2022). Cancer statistics, 2022. CA Cancer J. Clin..

[15] Smith A., Crouch S., Lax S., Li J., Painter D., Howell D., Patmore R., Jack A., Roman E. (2015). Lymphoma incidence, survival and prevalence 2004–2014: Sub-type analyses from the UK’s Haematological Malignancy Research Network. Br. J. Cancer.

[16] Statistical Databases on Incidence of Stroke and Myocardial Infarction in Sweden. https://www.socialstyrelsen.se/en/statistics-and-data/statistics/statistical-databases/.

[17] De Stefano V., Sorà F., Rossi E., Chiusolo P., Laurenti L., Fianchi L., Zini G., Pagano L., Sica S., Leone G. (2005). The risk of thrombosis in patients with acute leukemia: Occurrence of thrombosis at diagnosis and during treatment. J. Thromb. Haemost..

[18] McCarthy C.P., Steg G., Bhatt D.L. (2017). The management of antiplatelet therapy in acute coronary syndrome patients with thrombocytopenia: A clinical conundrum. Eur. Heart J..

[19] Falanga A., Leader A., Mbaglio C., Bagoly Z., Castaman G., Elamaly I., Lecumberri R., Niessner A., Pabinger I., Szmit S. (2022). EHA Guidelines on Management of Antithrombotic Treatments in Thrombocytopenic Patients With Cancer. HemaSphere.

[20] Canto J.G., Kiefe C.I., Rogers W.J., Peterson E.D., Frederick P.D., French W.J., Gibson C.M., Pollack C.V., Ornato J.P., Zalenski R.J. (2011). Number of coronary heart disease risk factors and mortality in patients with first myocardial infarction. JAMA.

